# How strong was the bottleneck associated to the peopling of the Americas? New insights from multilocus sequence data

**DOI:** 10.1590/1678-4685-GMB-2017-0087

**Published:** 2018

**Authors:** Nelson J.R. Fagundes, Alice Tagliani-Ribeiro, Rohina Rubicz, Larissa Tarskaia, Michael H. Crawford, Francisco M. Salzano, Sandro L. Bonatto

**Affiliations:** 1 Departamento de Genética, Universidade Federal do Rio Grande do Sul (UFRGS), Porto Alegre, RS, Brazil; 2 Fertilitat Centro de Medicina Reprodutiva, Centro Clínico da Pontifícia Universidade Católica do Rio Grande do Sul (PUCRS), Porto Alegre, RS, Brazil; 3 Division of Public Health Sciences, Fred Hutchinson Cancer Research Center, Seattle, WA, USA; 4 Laboratory of Biological Anthropology, University of Kansas, Lawrence, KS, USA; 5 Faculdade de Biociências, Pontifícia Universidade Católica do Rio Grande do Sul (PUCRS), Porto Alegre, RS, Brazil

**Keywords:** Isolation with Migration model, coalescent analysis, native Siberians

## Abstract

In spite of many genetic studies that contributed for a deep knowledge about the peopling of the Americas, no consensus has emerged about important parameters such as the effective size of the Native Americans founder population. Previous estimates based on genomic datasets may have been biased by the use of admixed individuals from Latino populations, while other recent studies using samples from Native American individuals relied on approximated analytical approaches. In this study we use resequencing data for nine independent regions in a set of Native American and Siberian individuals and a full-likelihood approach based on isolation-with-migration scenarios accounting for recent flow between Asian and Native American populations. Our results suggest that, in agreement with previous studies, the effective size of the Native American population was small, most likely in the order of a few hundred individuals, with point estimates close to 250 individuals, even though credible intervals include a number as large as ~4,000 individuals. Recognizing the size of the genetic bottleneck during the peopling of the Americas is important for determining the extent of genetic markers needed to characterize Native American populations in genome-wide studies and to evaluate the adaptive potential of genetic variants in this population.

## Introduction

Despite many scientific efforts have been made to unveil the peopling of the Americas, several important questions are still elusive (see Salzano, 2007; Goebel *et al.*, 2008; González-José and Bortolini, 2011; O’Rourke, 2011; Marangoni *et al.*, 2014 for recent reviews). Since the classic tripartite hypothesis for the origin of Native Americans proposed by Greenberg *et al.* (1986), a range of migration theories have been put forward to account for the linguistic, genetic, and morphologic diversity of human populations in the New World (see Marangoni *et al.*, 2014). Concerning genetic data, the analysis of uniparental markers have shown that most genetic diversity in Native Americans derives from a major population expansion after the Last Glacial Maximum (LGM) from an ancestral Beringian population (Zegura *et al.*, 2004; Tamm *et al.*, 2007; Achilli *et al.*, 2008, 2013; Fagundes *et al.*, 2008; Mulligan *et al.*, 2008; Bisso-Machado *et al.*, 2011, 2012; Mulligan and Szathmàry, 2017), but also that a single “migration wave” was too simplistic to account for the distribution of rare lineages, especially in North America, in agreement to the wide morphological variation found in Native American populations (*e.g,.* González-José *et al.*, 2008). Genomic studies based on a wide set of genetic markers have confirmed and extended this finding. A formal model choice procedure based on 401 microsatellite loci found that a model including recurrent gene flow between Siberian and Native American populations provided a better fit to the data compared to a model without gene flow (Ray *et al.*, 2010). In qualitative agreement with this finding, studies based on hundreds of thousands of SNP markers have consistently find evidence of ancient migration links between Native American and other Old World populations (Reich *et al.*, 2012; Raghavan *et al.*, 2014, 2015; Rasmussen *et al.*, 2015; Skoglund *et al.*, 2015).

An important parameter in population genetic studies is the effective population size, which can be broadly defined as the size of a simple Wright-Fisher population that undergoes the same amount of random drift as the actual population considered (Charlesworth, 2009). Characterizing the effective population size is instructive for understanding the selection-drift balance – which determines if nearly-neutral alleles behave as deleterious, neutral or adaptive – and of the size of linkage blocks – which is important in mapping studies (Hartl and Clark, 2007; Charlesworth, 2009). Genomic scans for genetic variation in humans have consistently shown that Native American populations are usually the least diverse in the globe (e.g., Prugnolle *et al.*, 2005; Li *et al.*, 2008), but these same studies find a very good correlation between genetic diversity and distance from East Africa. Therefore, the small genetic diversity in Native American populations could simply result from their long distance from East Africa. However, some colonization events may amplify the loss of genetic diversity if they are accompanied by a strong genetic bottleneck, as was probably the case for the huge differences in genetic variation levels between African and non-African populations (e.g., Yu *et al.*, 2002; Long *et al.*, 2009). Was this the case for Native Americans?

The first quantitative approach to infer the effective population size of the founder Native American population was developed by Hey (2005), who did a meta-analysis of nine sequence loci, used a likelihood-based inference and assumed a isolation with migration (IM) population model to suggest an extreme population bottleneck with an effective population size of ~70 individuals. Since this pioneer work, other groups tried to replicate this result using multilocus autosomal data, with partial success. Kitchen *et al.* (2008) re-analyzed Hey’s dataset, adding mtDNA genomic data under different priors for migration rates and suggested an effective population size ranging from 1,000 to 5,400 individuals. Ray *et al.* (2010), using a dataset of 401 STRs, estimated an effective founder population size between 42 and 140 individuals (with a median of 87 individuals). Between these two extremes, Fagundes *et al.* (2007), based on the re-sequencing of 50 short loci, estimated an effective founder size of ~450 individuals (with a 95% credible interval (CI) ranging from 71 to 1,280 individuals). Recent autosomal data generated from admixed Latino populations also provided very different figures. Gutenkunst *et al.* (2009), based on a very large dataset of more than 13,000 SNPs, suggested a value of 800 effective individuals, with a confidence interval between 140 and 1,600 individuals; while Wall *et al.* (2011), using resequencing data, estimated a bottleneck effective population size not larger than 150 individuals. Gravel *et al.* (2013) proposed intermediate values of about 514 effective individuals, ranging between 316 and 2,264 individuals.

In this study we generated DNA sequence data from Native American and Siberian individuals for nine autosomal loci totaling about ~17.5 kb/ individual. We also included data from other Asian individuals and used an isolation-with-migration population model to study the pattern of population subdivision and to estimate the effective population size of the first Native American settlers. To our knowledge, this is the first time that this parameter is explicitly estimated using a common set of individuals from Native American populations typed for autosomal sequence data and analyzed under a full-likelihood method. Overall, our results confirm a late Pleistocene split between Siberians and Native Americans, with Asian populations splitting off some thousand years earlier. Our results also corroborate the idea that the Native American founder population underwent a strong bottleneck, though less extreme than previously suggested.

## Material and Methods

### Samples and ethics statement

We selected DNA samples from 10 Native American individuals scattered across Central and South America, representing several different tribal affiliations. More specifically, we used DNA samples of one individual from each of the following populations: Aché (Paraguay), Arara (Brazil), Bribri (Costa Rica), Guatuso (Costa Rica), Guaymi (Costa Rica), Lengua (Argentina), Quechua (Peru), Waiwai (Brazil), Xavante (Brazil), and Zoró (Brazil). The same sampling scheme was applied to Siberian populations, and one individual from each of the following populations was studied: Altai, Aleut, Buryat, Chukchi, Evenki, Even, Itel’men, Kalmyk, Koryak, and Tuva. For a more thorough characterization of the genetic diversity in Asia, 15 individuals from China genetically characterized by Frisse *et al.* (2001) have been included in the final dataset.

For Native American participants, ethical approval was provided by the Brazilian National Ethics Commission (CONEP Resolution no. 123/98), according to all the ethic practices required at the time. Individual and tribal informed oral consent was obtained from all participants, since they were illiterate, and were obtained according to the Helsinki Declaration. Record was made of the Amerindian leaders and National Indian Foundation (FUNAI) officials consents. The ethic committee approved the oral consent procedure, as well as the use of these samples in population and evolutionary studies. The samples from Siberian populations were collected following the collapse of the Soviet Union. Only verbal informed consent was obtained. This form of consent was given with witnesses present. The verbal informed consent was necessary because of the association of signing documents to political confessions during the days of the USSR. Both the University of Kansas Institutional Review Board and NSF approved this alternative method of informed consent.

### Molecular markers and methods

We studied nine noncoding autosomal regions first investigated by Frisse *et al.* (2001). They correspond to regions 1-5 and 7-10 characterized in the indicated study; and each are about 10 kbp in length, for which 1,000 bp at each end was sequenced. This approach has the advantage to detect possible effects of recombination as there is some distance between the edges of each marker. Following the above-indicated authors (Frisse *et al.*, 2001), each of these two-segment units will be referred as a “locus pair”. These regions have been also used in other studies (Voight *et al.*, 2005; Wall *et al.*, 2008; Scliar *et al.*, 2012).

Genomic DNA of all samples was initially subjected to a whole genomic amplification (WGA) using GenomePhi (GE Healthcare), a strategy that is considered adequate for subsequent downstream procedures such as PCR and sequencing (El Sharawy *et al.*, 2012). We then used the WGA product diluted 10x as template for the specific PCR amplifications. For each of the nine regions we designed external and internal primer sets to allow for PCR amplification and sequencing by the Sanger method. The amplification of PCR products were checked in agarose gel stained with GelRed^TM^, and purified with polyethylene glycol (Dunn and Blattner, 1987), after which they were subjected to automated sequencing in a MegaBACE 1000 machine (GE Helthcare) using the manufacturer’s kits and protocols.

### Data analysis

Sequences were assembled using PhredPhrap (Ewing *et al.*, 1998) and visualized in Consed (Gordon *et al.*, 1998) using reference sequences obtained from GenBank to guide the assembly. Heterozygous positions were easily identified by visual inspection. All positions containing singletons were confirmed using independent PCR and sequencing reactions. Haplotypes for each locus were estimated using PHASE 2.1 (Stephens *et al.*, 2001) using five independent runs to check for consistency and convergence.

Basic genetic diversity measures, such as haplotype and nucleotide diversity, neutrality tests (Tajima’s D and Fu’s F_S_), and measures based on F-statistics were performed in the Arlequin 3.5 program (Excoffier and Lischer, 2010). The null hypothesis of intralocus no recombination was evaluated in the DnaSP 5 software (Librado and Rozas, 2009) using the ZZ statistic (Rozas *et al.*, 2001). For each locus, the substitution rate was estimated under the assumption of a lognormal relaxed molecular clock (Drummond *et al.*, 2006) and assuming for the human-chimpanzee divergence a normal distribution with mean of 6.5 million years (e.g., Macaulay *et al.*, 2005) and standard deviation of 0.3 million years. Substitution rate estimates were performed in the Beast 1.6.5 program (Drummond and Rambaut, 2007). For all loci, the HKY+G+I evolutionary model was assumed with parameters allowed to vary freely.

Two alternative demographic assumptions were tested; first a constant population size model and Bayesian skyline demographic model, in which the gene genealogy of each locus is divided in “epochs” that can have different population sizes (Drummond *et al.*, 2005). The demographic model providing the best fit with the data was selected using Bayes Factors (Kass and Raftery, 1995) estimated in Tracer 1.5 (http://beast.bio.ed.ac.uk/Tracer), which in all cases supported the constant population size model (data not shown). Each analysis was run for 100,000,000 generations, sampling every 1,000 generations, and the first 10% samples were discarded as burn-in.

Demographic parameters were estimated under the isolation-with-migration population model (*IM*), as implemented in the IM program (Hey, 2005). In short, the model assumes that moving forward in time, an ancestral population of size *θ*_A_ splits in two sister populations at time T according to parameter *s*, which varies from 0 to 1. Thus, one of the descendant populations has a founder population size of *θ*_A_ *s*, while the remaining population has a founder size of *θ*_A_(1 – *s*). After the split, the two populations are allowed to grow or shrink and they may exchange migrants in an asymmetric way.

Because the migration parameter affects the estimates for the founder population sizes (Kitchen *et al.*, 2008), we used two migration scenarios, the first one assuming that no migration took place after the population split, and the second one assuming a maximum migration value estimated from contemporary European populations, as in Kitchen *et al.* (2008). The lower limit for population split was set at 15 thousand years ago (kya), based on the archeological record for the Americas, for which some of the oldest sites includes the well accepted Monte Verde, in southern Chile, dated at 14,500 years ago, and Swan Point, in Central Alaska, dated at 14,000 years ago (Goebel *et al.*, 2008). The analysis was run for 5,000,000 steps sampling every 100 steps. Consistency was checked by running the same settings multiple times using different seeds. To ensure the quality of the estimates, the effective sample size (ESS) for all parameters in all scenarios was higher than 500.

## Results and Discussion

### General results and SNP distribution

The full alignment of all nine regions in Chinese, Siberian, and Native American samples produced a data matrix consisting of 17,456 bp and 66 SNPs. All generated sequences are available in GenBank under accession numbers KF468820-KF469176. Chinese was the population with the highest number of SNPs, followed by Native American and Siberian (49, 39 and 36, respectively). When Chinese and Siberian samples are merged into an “Asian” metapopulation, the number of SNPs rises to 62, suggesting that these two subgroups are genetically distinct, with Siberians having 13/36 SNPs that were not found in Chinese. The Native American sample had four private SNPs, which were not shared with either Chinese or Siberian samples.

### Basic population genetic quantities

Average values for several common population genetics statistics, together with their standard deviations over loci are presented in Table 1. Similar tables for each locus are available in the Table S1 (Supplementary material). In general, observed heterozygosity (*H*_OBS_) was lower than expected heterozygosity (*H*_EXP_), which may be related to the Wahlund effect, that is, a deficit in observed heterozygosity caused by population structure (Hartl and Clark, 2007). This is expected from the sampling scheme adopted in this study, in which, for most populations, we sampled genetics lineages from a single individual from different local populations. In line with this reasoning, the smallest difference between *H*_OBS_ and *H*_EXP_ was found for the Chinese, which is the geographically more homogeneous group, even though local inbreeding may also play a role in lowering *H*_OBS_. Even though the Siberian showed the lowest genetic diversity in general, nucleotide diversity (*π*) was lowest in the Chinese, despite the relative high number of haplotypes and polymorphic sites. These observations are compatible with a recent Han population expansion (Zheng *et al.*, 2011), which would increase the number of haplotypes and polymorphic sites due to the maintenance of new, rare mutations that will have few impact over *π*. This is in agreement with the results of neutrality statistics considering that a population expansion would drive these statistics towards negative values. For both Tajima’s D (TajD) and Fu’s F_S_, the Chinese population is the one with the lowest average scores, even though individual tests are barely statistically significant at *P* < 0.05 for TajD and *P* < 0.02 for F_S_, probably due to the limited power of these tests considering the limited sample size available. On the other hand, for both neutrality statistics, Native Americans have the largest average values, in agreement with a possible genetic bottleneck during the early settlement of the Americas.

**Table 1:** Average genetic diversity statistics over loci. Standard deviation values are shown in parentheses and are calculated over loci^1^.

Population	Average no. hapl.	Total S	S	Gene div	*H*_OBS_	*H*_EXP_	π(%)	TajD	Fu’s F_S_
Asian	7.33 (3.43)	62	6.89 (3.26)	0.638 (0.218)	0.529 (0.205)	0.717 (0.187)	0.072 (0.029)	0.295 (1.049)	-0.919 (2.825)
Chinese	5.44 (2.40)	49	5.44 (2.83)	0.544 (0.233)	0.514 (0.255)	0.586 (0.180)	0.054 (0.027)	0.062 (1.207)	-0.528 (1.920)
Siberian	3.67 (1.67)	36	4.00 (2.50)	0.536 (0.217)	0.556 (0.218)	0.679 (0.278)	0.075 (0.040)	0.444 (0.814)	0.706 (1.032)
Native American	3.78 (1.09)	39	4.33 (2.18)	0.599 (0.097)	0.502 (0.153)	0.801 (0.163)	0.077 (0.029)	0.616 (0.926)	1.081 (1.634)
Overall	8.22 (3.83)	66	7.33 (3.46)	0.674 (0.182)	0.521 (0.154)	0.790 (0.162)	0.082 (0.023)	0.743 (1.077)	-0.630 (3.520)

*Note:*

1 Average no. hapl: average number of haplotypes; total S: total number of S over loci; S: segregating sites; Gene div: Gene diversity; *H*_OBS_: Observed heterozygosity; *H*_EXP_: Expected heterozygosity; π: nucleotide diversity; TajD: Tajima’s D.

Pairwise Φ_ST_ values show Siberians closer to the Chinese (Table 2). However, from a locus-by-locus perspective, Siberians are “intermediate” between Chinese and the Native Americans, since for all but one locus Siberians show non-significant Φ_ST_ values with one (Chinese, three loci; Native Americans, one loci), or both (four loci) populations (Table S2). Native Americans and Chinese represent the most divergent population pair. This was expected given their more distant geographic relationship and considering recent models for the peopling of the Americas that suggest that the ancestral population of Native Americans had ancestry from both East and West Eurasians (Raghavan *et al.*, 2014). In addition, some sort of secondary genetic contact between Native American and Siberian populations may help to explain the observed pattern (e.g., González-José *et al.*, 2008; Azevedo *et al.*, 2011; Ray *et al.*, 2010; Reich *et al.*, 2012; Raghavan *et al.*, 2015). AMOVA results are very similar irrespective of considering three (Chinese *vs.* Siberian *vs.* Native American) or two (Chinese + Siberian *vs.* Native American) populations, with the among population component explaining 18.13% or 19.80% of the total genetic variance, respectively.

**Table 2:** Average pairwise Φ_ST_ (lower diagonal) and their standard deviations over loci (upper diagonal).

Population	Asian	Chinese	Siberian	Native American
Asian	-	0.0198	0.1108	0.2039
Chinese	-0.0128	-	0.1791	0.2315
Siberian	0.0199	0.0713	-	0.2119
Native American	0.1963	0.2284	0.1641	-

### Mutation and recombination

For all nine loci, the constant population size model provided a better fit to the results (data not shown) and, therefore, this model was used for the estimation of the overall evolutionary rate. Substitution rates per site for each locus are presented in (Table S3), and varied from 5.59x10^-10^ substitutions/site/year (s/s/y) to 1.38x10^-9^ s/s/y, with an average value of 9.61x10^-10^ s/s/y, which is close to previous estimates for autosomes (Fagundes *et al.*, 2007). For all loci, the null hypothesis of no recombination could not be rejected (*P* > 0.05; Table S1). These results suggest that eventual recombination events, if any, affecting this dataset have been weak enough to violate the assumption of no recombination among independent loci in the IM models.

### IM scenarios

Results for IM scenarios for different population pairs are presented in Table 3. In general, the effective size of the ancestral population (N_A_) were estimated within a narrow credible interval in all comparisons. However, most parameters for current effective population sizes had broad credible intervals (Figures S1-S4). Including or not migration resulted in very similar estimates for all parameters (Table 3; Figures S1-S11), and thus we will only discuss the results based on the “full migration” scenarios. Importantly, even though our data is not informative for precise estimates of the migration parameters directly, resulting in flat posterior densities (Figure S5), maintaining migration in the analysis allows estimating the effective size of the founder population of the Americas while accounting for the impact and uncertainty of gene flow estimates (González-José *et al.*, 2008; Azevedo *et al.*, 2011; Ray *et al.*, 2010; Reich *et al.*, 2012; Raghavan *et al.*, 2015; Skoglund *et al.*, 2015).

**Table 3:** Values for the Isolation with Migration scenarios tested. The 95% credible interval is shown in parentheses^1^.

Populations	Migr.	N_1_	N_2_	N_A_	Time (y)	Founder Pop2	M_1-2_	M_2-1_
		(95% CI)	(95% CI)	(95% CI)	(95% CI)	(95% CI)	(95% CI)	(95% CI)
1. Asian vs. 2. Nat. American	No	13,237	1,309	6,255	15,436	229		
		(8,582 - 277,094)	(1,018 - 273,313)	(4,509 - 9,455)	(15,133 - 29,479)	(144 - 3,165)	-	-
	Yes	7,127	1,018	6,255	15,436	229	3x10^-4^	1x10^-4^
		(5,091 - 274,476)	(727 - 267,494)	(4,509 - 9,746)	(15,194 - 37,651)	(123 - 3,409)	(~0.00 - 3x10^-4^)	(~0.00 - 3x10^-4^)
1. Chinese vs. 2. Nat. American	No	5,171	1,625	6,353	24,334	233		
		(2,807 - 254,865)	(1,034 - 247,182)	(4,284 - 9,899)	(15,799 - 38,862)	(167 - 3,638)	-	-
	Yes	4,284	1,330	6,353	25,787	233	0.00	0.00
		(2,216 - 253,683)	(738 - 245,705)	(4,284 - 10,195)	(15,678 - 39,164)	(125 - 3,549)	(~0.00 - 3x10^-4^)	(~0.00 - 3x10^-4^)
1. Chinese vs. 2. Siberian	No	6,937	3,690	5,756	18,886	416		
		(3,395 - 263,752)	(2,509 - 279,987)	(3,985 - 9,298)	(15,436 - 37,288)	(233 - 3,292)		-
	Yes	5,166	3,395	5,756	19,491	387	0.00	1x10^-4^
		(2,804 - 255,487)	(2,214 - 279,397)	(3,985 - 9,298)	(15,557 - 38,499)	(167 - 3,132)	(~0.00 - 3x10^-4^)	(~0.00 - 3x10^-4^)
1. Siberian vs. 2. Nat. American	No	3,540	1,713	5,139	15,436	300		
		(2,169 - 218,672)	(1,484 - 216,160)	(3,540 - 8,564)	(15,133 - 29,419)	(200 - 2,954)	-	-
	Yes	1,484	1,484	5,367	15,436	300	1x10^-4^	3x10^-4^
		(1,028 - 216,388)	(1,028 - 215,475)	(3,540 - 8,792)	(15,133 - 34,685)	(113 - 2,954)	(~0.00 - 3x10^-4^)	(~0.00 - 3x10^-4^)

*Note:*

1 N_1_: effective population size of population 1; N_2_: effective population size of population 2; N_A_: effective population size of the ancestral population; Time (y): Time in years; Founder Pop2: Effective population size of the founders of population 2; M_1-2_: Migration rate (backwards) from population 1 to 2 M_2-1_: Migration rate (backwards) from population 2 to 1. All effective population sizes are in number of individuals.

**Figure 1:**
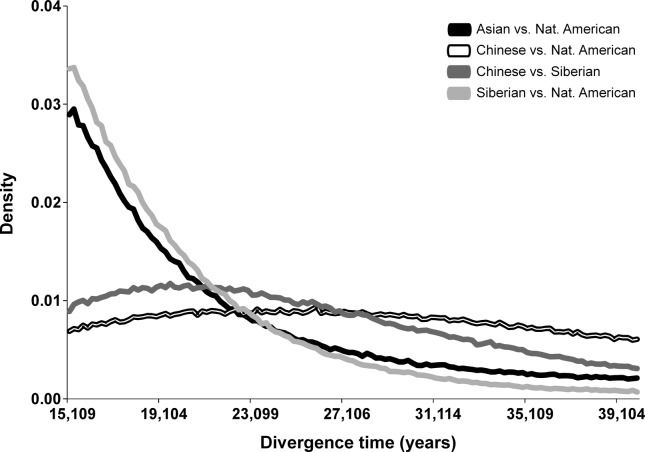
Posterior densities for divergence time (years) in all scenarios tested including migration. Divergence between Asian and Native American is shown in solid black, between Chinese and Native American in white with black contour, between Chinese and Siberian in dark gray, and between Siberian vs. Native American in light gray, as shown in the graphical legend.

Divergence time estimates showed two distinct patterns (Figure 1; Figure S6). Whenever Siberians are included in the comparison against Native Americans (either as a single population or as part of an “Asian” metapopulation), the time of divergence goes toward the lower limit set by the prior at 15 kya. Even though the IM model should be able to separate the effects of divergence and migration, the relatively small sample sizes and the overall genetic similarity between these groups make difficult distinguishing between recent migration and shared ancestry. Alternatively, this result may reflect a genuine impact of recent migration between these groups, even though recent migration is thought as having a weaker impact on Central and South Amerindians compared to North Amerindians (González-José *et al.*, 2008; Azevedo *et al.*, 2011; Ray *et al.*, 2010; Reich *et al.*, 2012; Raghavan *et al.*, 2015). On the other hand, whenever Chinese are contrasted with Native Americans or Siberians the divergence time parameter shows roughly flat posteriors, with point estimates around 25 kya or 19 kya *vs.* Native Americans or Siberians, respectively, in agreement with a more recent shared ancestry between Siberians and Native Americans (Raghavan *et al.*, 2015) compared to Han, even though contemporary Siberians lack the Western Eurasian ancestry component represented by the Mal’ta individual (Raghavan *et al.*, 2014).

**Figure 2:**
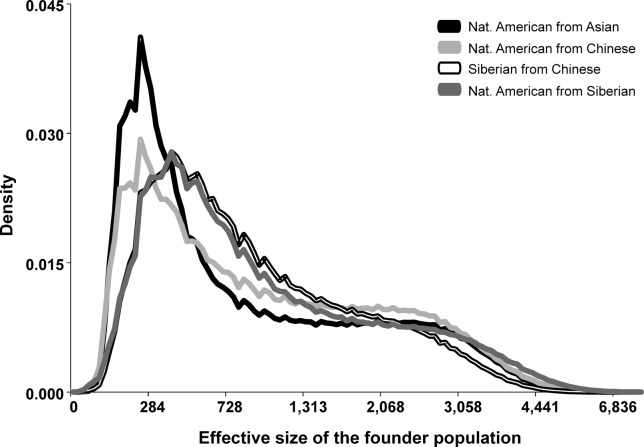
Posterior densities for the effective sizes of the founder population. The size of the founder population for Native American from Asia is shown in solid black, for Native American from Chinese in light gray, for Siberian from Chinese in white with black contour, and for Native American from Siberian in dark gray, as shown in the graphical legend.

Estimates of the splitting parameter *s* resulted in heavier densities around small values, suggesting, for all scenarios, a reduction on Native American effective population size compared with Asian, Chinese and Siberian populations, as well as another population bottleneck for the Siberian population when compared against the Chinese (Figure S7). The effective population size of the founder populations (parameter Founder Pop2 in Table 3) is the product between the effective size of the ancestral population and the splitting parameter *s*. The posterior densities for all scenarios are presented in Figures S8-S11, and are very similar for scenarios including or not migration. Considering scenarios with migration (Figure 2), the effective size of the founding population for Native Americans was estimated around 229 (*vs.* Asians), 233 (*vs.* Chinese), or 300 individuals (*vs.* Siberian), with 95% credible intervals between ~100 – 3,700 individuals. It should be noted, however, that even though the confidence intervals are wide (Table 3), the density is asymmetrical, with much of the posterior probability falling closer to the smallest values (Figure 2). For example, in the case of the Asian vs. Native American comparison, the 50% highest posterior density falls within a range of small values (between 123-587 individuals), suggesting that small values are more likely than larger ones. These values represent intermediate estimates between the extreme bottleneck scenario proposed by Hey (2005), and the larger numbers estimated by Kitchen *et al.* (2008). Our results also show some evidence of a genetic bottleneck during the divergence of Siberian populations from their Asian (Chinese) ancestors (Figure 2), but while such reduction may have been milder than that associated to the peopling of the Americas, the credible intervals for these estimates are broad.

**Figure 3:**
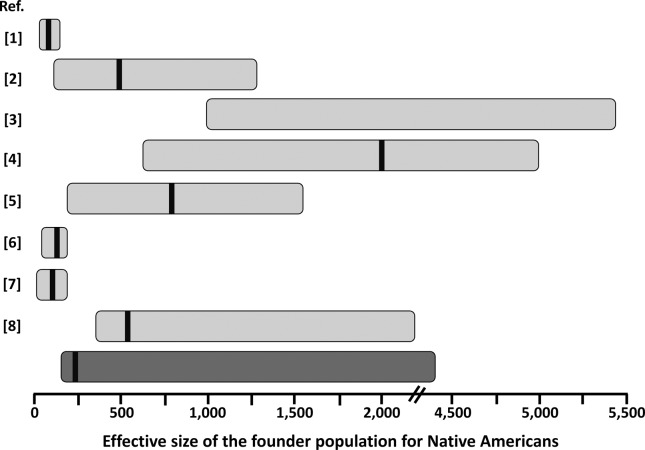
Schematic representation of estimates of the founder population size for Native Americans from this (dark gray bar) and previous (light gray bars) studies. The horizontal bars represent the approximate 95% credible interval for the published values. Point estimates, when reported, are represented by the black vertical bar within the horizontal bars. Please note the discontinuity in the *x*-axis between 2,000 and 4,500. References (Ref.) are: 1 – Hey (2005); 2 – Fagundes *et al.* (2007); 3 – Kitchen *et al.* (2008); 4 – Fagundes *et al.* (2008); 5 – Gutenkunst *et al.* (2009); 6 – Ray *et al.* (2010); 7 – Wall *et al.* (2011); 8 – Gravel *et al.* (2013). The values presented in Hey (2005) were recalculated based on a generation time of 25 years.

Our estimates for the effective size of the founder population of Native (Central and South) Americans are in good agreement with those reported for other autosomal markers (Figure 3), except for the original estimates of Hey (2005) which are smaller, probably due to the use of an unconstrained prior on migration rate, as suggested by Kitchen *et al.* (2008). On the other hand, estimates including complete mtDNA genomes (Fagundes *et al.*, 2008; Kitchen *et al.*, 2008) are usually larger (Figure 3). This may be due to a larger effective population size for women. Further analysis of X-chromosome and Y-chromosome data would help to clarify this issue. Interestingly, our estimates were comparable with those based on studies using admixed populations from a restricted geographic area (Gutenkunst *et al.*, 2009; Wall *et al.*, 2011; Gravel *et al.*, 2013), suggesting that Latino populations may be extremely valuable sources of information on Native American history, as have been shown for inferences on extinct Native American ethnicities (Marrero *et al.*, 2007). Estimating the effective size of the Native American founder population is important in medical genetic approaches, as in the case of estimating the average size of linkage disequilibrium blocks and how many genetic markers (e.g. SNPs) will be effective for gene-disease mapping in this (or derived) population (e.g., Wall *et al.*, 2011). This parameter is also of crucial importance to understand the fate of adaptive alleles in the founder population of Native Americans, that might behave as neutral depending on the effective population size (Ohta, 1992). A relatively strong reduction in effective size in the founder population of Native Americans might explain why some possibly adaptive genetic variants in other populations do not show any signature of selection in the Americas (e.g*.,* Paixão-Côrtes *et al.*, 2011; Augusto *et al.*, 2013).

Our study used a relatively small sample size to estimate the effective size of the founder population. Felsenstein (2006) suggested that a small number of individuals (n ≤ 8) may be sufficient for estimating the effective population size, because most of each gene genealogy would be known with a limited number of genetic lineages, provided that a sufficient number of independent genealogies were studied. Therefore, this number seems appropriate for a broad characterization of the effective size of the founder population, even though it is certainly very small to thoroughly characterize the genetic diversity of these populations at these loci. Other interesting questions regarding the peopling of the Americas, such as differences in the effective population size for different regions within the New World, would certainly require a much larger sample size. As discussed previously, it is noteworthy that our results provided similar results compared to other autosomal-based studies, even when only local admixed populations were sampled.

One of the major statistical advantages of the IM model is that it can use the full dataset in a maximum likelihood framework, which increase the power of evolutionary demographic parameter estimation compared to techniques such as approximate Bayesian computation (Beaumont, 2008; Nielsen and Beaumont, 2009). Such higher statistical power, however, may come at the expense of some biological realism. For example, scenarios suggesting gene flow between Asia and America usually assume that gene flow had a late start compared to the initial population subdivision (González-José *et al.*, 2008; Ray *et al.*, 2010). Unfortunately it is not possible yet to implement such specific constrains within the full likelihood framework of the IM models (Nielsen and Beaumont, 2009). Interestingly, in the present analysis including or not including migration did not result in major differences for any demographic parameter. Migration would reduce the estimates for the founder population size in the context of the peopling of the New World (Kitchen *et al.*, 2008). This is intuitive, since migration would lead to new genetic diversity coming to the continent, thus resulting in a smaller population size estimate for the initial founding event. The recent discussions on the importance of secondary migration to account for the morphological and genetic diversity of Native Americans (González-José *et al.*, 2008; Azevedo *et al.*, 2011; Ray *et al.*, 2010; Reich *et al.*, 2012; Raghavan *et al.*, 2015; Skoglund *et al.*, 2015; von Cramon-Taubadel *et al.*, 2017) indicate the need to add a further step on the traditional three-stage model (Mulligan and Szathmàry, 2017).

## Supplementary material

The following online material is available for this article:

Table S1 - Genetic diversity statistics for each locus.

Table S2 - Pairwise Φ_ST_ for each locus.

Table S3 - Estimated substitution rates (μ) for each locus.

Figure S1 - Posterior density of effective population sizes for Asian vs. Native Americans.

Figure S2 - Posterior density of effective population sizes for Chinese vs. Native Americans.

Figure S3 - Posterior density of effective population sizes for Chinese vs. Siberians.

Figure S4 - Posterior density of effective population sizes for Siberian vs. Native Americans.

Figure S5 - Posterior density for migration parameters for all scenarios.

Figure S6 - Posterior density for divergence times (in years) for all scenarios.

Figure S7 - Posterior density for the splitting parameter s for all scenarios.

Figure S8 - Posterior density for the effective size of the founder population for Native Americans, from Asia, with or without migration.

Figure S9 - Posterior density for the effective size of the founder population for Native Americans, from Chinese, with or without migration.

Figure S10 - Posterior density for the effective size of the founder population for Siberians, from Chinese, with or without migration.

Figure S11 - Posterior density for the effective size of the founder population for Native Americans, from Siberians, with or without migration.
