# Supplementary material for: How strong was the bottleneck associated to the peopling of the Americas? New insights from multilocus sequence data
Source: Genet Mol Biol. 2018;41(1 Suppl 1):206–14. doi: 10.1590/1678-4685-GMB-2017-0087 (PMC5913727; doi:10.1590/1678-4685-GMB-2017-0087)
Supplement: Supplementary file 1 [file 1415-4757-GMB-41-01-2017-0087-s012.pdf]

Supplementary Material to "How strong was the bottleneck associated to the peopling of the Americas? New insights from multilocus sequence data"

Table S1 – Genetic diversity statistics for all loci individually. Standard deviation values are shown in parentheses.

Locus	Population	n	Nº Hapl	S	Gene Div	H_{OBS}	H_{EXP}	π %	TajD	Fu's F_S	ZZ test
Locus1	Asian	44	10	10	0.826 (0.032)	0.591	0.849	0.062 (0.043)	-0.725	-3.547*	-
	Chinese	32	8	9	0.746 (0.055)	0.625	0.750	0.051 (0.038)	-1.040	-2.739*	-
	Siberian	12	2	1	0.409 (0.133)	0.500	0.712	0.018 (0.020)	0.541	0.735	-
	Native American	18	4	3	0.549 (0.126)	0.222	0.758	0.027 (0.026)	-0.026	-0.668	-
	Overall	62	12	11	0.848 (0.021)	0.484	0.890	0.070 (0.048)	-0.487	-4.646*	0.168^{NS}
Locus2	Asian	48	7	5	0.272 (0.084)	0.250	0.308	0.062 (0.051)	-0.220	-3.007*	-
	Chinese	32	6	4	0.292 (0.104)	0.250	0.345	0.061 (0.052)	-0.522	-2.449	-
	Siberian	16	3	4	0.242 (0.135)	0.250	0.242	0.067 (0.057)	-0.654	0.586	-
	Native American	20	4	5	0.574 (0.090)	0.500	0.574	0.101 (0.074)	-0.206	0.495	-
	Overall	68	9	6	0.388 (0.074)	0.324	0.410	0.077 (0.059)	-0.158	-3.854*	-0.048^{NS}
Locus3	Asian	48	7	9	0.815 (0.027)	0.708	0.823	0.069 (0.055)	0.579	2.595	-
	Chinese	32	4	3	0.673 (0.048)	0.688	0.673	0.074 (0.058)	0.572	0.285	-
	Siberian	16	3	2	0.608 (0.90)	0.750	0.683	0.061 (0.053)	0.661	0.415	-
	Native American	20	3	2	0.658 (0.065)	0.700	0.916	0.074 (0.060)	1.486	1.033	-
	Overall	68	7	9	0.825 (0.019)	0.706	0.892	0.069 (0.055)	0.748	3.933	-0.080^{NS}
Locus4	Asian	48	7	6	0.731 (0.036)	0.750	0.843	0.082 (0.052)	1.303	0.336	-
	Chinese	32	6	6	0.702 (0.059)	0.688	0.702	0.073 (0.048)	0.627	0.210	-

Locus	Population	n	Nº Hapl	S	Gene Div	H_{OBS}	H_{EXP}	π %	TajD	Fu's F_S	ZZ test
	Siberian	16	5	6	0.650 (0.108)	0.875	0.975	0.090 (0.059)	0.757	0.572	-
	Native American	20	5	6	0.700 (0.082)	0.600	0.979	0.076 (0.050)	0.324	0.494	-
	Overall	68	8	7	0.792 (0.021)	0.706	0.918	0.099 (0.060)	1.741	0.829	0.123^{NS}
Locus5	Asian	48	15	11	0.752 (0.56)	0.667	0.864	0.075 (0.047)	-0.095	-6.455**	-
	Chinese	32	10	8	0.694 (0.080)	0.625	0.694	0.068 (0.044)	0.374	-2.922	-
	Siberian	16	7	8	0.842 (0.060)	0.750	0.983	0.090 (0.057)	0.196	-1.002	-
	Native American	20	5	8	0.695 (0.081)	0.500	0.932	0.067 (0.043)	-0.546	0.532	-
	Overall	68	16	12	0.729 (0.047)	0.618	0.884	0.072 (0.042)	-0,251	-6.528**	0.098^{NS}
Locus7	Asian	48	5	10	0.268 (0.082)	0.250	0.527	0.041 (0.030)	-1.427*	0.205	-
	Chinese	32	4	9	0.182 (0.090)	0.125	0.290	0.022 (0.020)	-2.212**	-0.665	-
	Siberian	16	3	7	0.425 (0.133)	0.500	0.833	0.075 (0.050)	-0.058	2.941	-
	Native American	20	3	6	0.542 (0.076)	0.600	0.921	0.111 (0.067)	2.476	4.885	-
	Overall	68	6	10	0.371 (0.067)	0.353	0.684	0.068 (0.044)	-0.261	1.346	0.196^{NS}
Locus8	Asian	48	4	2	0.614 (0.044)	0.583	0.742	0.108 (0.091)	1.084	0.005	-
	Chinese	32	4	2	0.542 (0.074)	0.563	0.542	0.092 (0.083)	0.495	-0.672	-
	Siberian	16	3	2	0.692 (0.058)	0.625	0.625	0.152 (0.124)	1.369	0.826	-
	Native American	20	3	2	0.616 (0.077)	0.300	0.868	0.113 (0.099)	0.611	0.475	-
	Overall	68	4	2	0.606 (0.038)	0.500	0.781	0.106 (0.089)	1.160	0.215	0.000^{NS}
Locus9	Asian	48	4	3	0.598 (0.054)	0.292	0.694	0.029 (0.028)	0.233	0.737	-
	Chinese	32	2	2	0.272 (0.089)	0.313	0.490	0.031 (0.029)	0.193	1.856	-
	Siberian	16	2	2	0.233 (0.126)	0.250	0.233	0.027 (0.027)	-0.578	1.127	-

Locus	Population	n	Nº Hapl	S	Gene Div	H_{OBS}	H_{EXP}	π %	TajD	Fu's F_S	ZZ test
	Native American	20	2	2	0.395 (0.101)	0.500	0.532	0.045 (0.038)	0.939	2.343	-
	Overall	68	4	3	0.707 (0.024)	0.353	0.767	0.051 (0.040)	1.875	2.225	0.000^{NS}
Locus10	Asian	48	7	6	0.778 (0.38)	0.667	0.807	0.118 (0.073)	1.924	0.859	-
	Chinese	32	5	6	0.790 (0.034)	0.750	0.790	0.129 (0.079)	2.072	2.346	-
	Siberian	16	5	4	0.725 (0.098)	0.500	0.825	0.094 (0.063)	1.759	0.159	-
	Native American	20	5	5	0.663 (0.95)	0.600	0.732	0.082 (0.057)	0.482	0.140	-
	Overall	68	8	6	0.803 (0.021)	0.647	0.882	0.123 (0.075)	2.325	0.808	0.040^{NS}

Note: n – sample size (number of chromosomes); Nº Hapl – number of haplotypes; S – segregating sites; Gene Div – Gene diversity; H_{OBS} – Observed heterozigosity; H_{EXP} – Expected heterozigosity; π – nucleotide diversity; TajD – Tajima's D. *Marginally significant values (0,10<P<0,05 for TajD; 0,05<P<0,02 for Fu's F_S); **Significant values (P<0,05 for TajD; P<0,02 for Fu's F_S). NS – Not significant values for the recombination test.
