# Supplementary material for: How strong was the bottleneck associated to the peopling of the Americas? New insights from multilocus sequence data
Source: Genet Mol Biol. 2018;41(1 Suppl 1):206–14. doi: 10.1590/1678-4685-GMB-2017-0087 (PMC5913727; doi:10.1590/1678-4685-GMB-2017-0087)
Supplement: Supplementary file 2 [file 1415-4757-GMB-41-01-2017-0087-s013.pdf]

Supplementary Material to "How strong was the bottleneck associated to the peopling of the Americas? New insights from multilocus sequence data"

Table S2 – Pairwise Φ_{ST} for each locus.

Locus	Population	Asian	Chinese	Siberian	Native American
Locus 1	Asian	-			
	Chinese	0.0373*	-		
	Siberian	0.3107*	0.5320*	-	
	Native American	0.3370*	0.5209*	0.3281*	-
Locus 2	Asian	-			
	Chinese	-0.0245	-		
	Siberian	-0.0345	-0.0288	-	
	Native American	0.1011*	0.0999*	0.0592	-
Locus 3	Asian	-			
	Chinese	-0.0245	-		
	Siberian	-0.0338	-0.0284	-	
	Native American	-0.0328	-0.0416	-0.0346	-
Locus 4	Asian	-			
	Chinese	-0.0097	-		
	Siberian	0.0198	0.0922	-	
	Native American	0.3704*	0.4531*	0.2308*	-
Locus 5	Asian	-			
	Chinese	-0.0178	-		
	Siberian	-0.0101	0.0274	-	
	Native American	-0.0086	0.0216	-0.0421	-
Locus 7	Asian	-			
	Chinese	-0.0089	-		

Locus	Population	Asian	Chinese	Siberian	Native American
	Siberian	0.0054	0.0785*	-	
	Native American	0.2295*	0.3133*	0.0395	-
Locus 8	Asian	-			
	Chinese	-0.0200	-		
	Siberian	-0.0191	0.0083	-	
	Native American	-0.0322	-0.0369	-0.0207	-
Locus 9	Asian	-			
	Chinese	-0.0263	-		
	Siberian	-0.0417	-0.0454	-	
	Native American	0.5459*	0.5157*	0.5351*	-
Locus 10	Asian	-			
	Chinese	-0.0208	-		
	Siberian	-0.0173	0.0059	-	
	Native American	0.2560*	0.2097*	0.3819*	-

Note: **P*<0.05
