# Supplementary material for: How strong was the bottleneck associated to the peopling of the Americas? New insights from multilocus sequence data
Source: Genet Mol Biol. 2018;41(1 Suppl 1):206–14. doi: 10.1590/1678-4685-GMB-2017-0087 (PMC5913727; doi:10.1590/1678-4685-GMB-2017-0087)
Supplement: Supplementary file 3 [file 1415-4757-GMB-41-01-2017-0087-s014.pdf]

# Supplementary Material to "How strong was the bottleneck associated to the peopling of the Americas? New insights from multilocus sequence data"

**Table S3 –** Estimated substitution rates (μ) for each locus.

| Locus | Nº sites | μ per site (95% CI) | μ per gene (95% CI) |
|---|---|---|---|
| 1 | 2328 | $1.38 \times 10^{-09}$ ($8.15 \times 10^{-10}$ - $2.08 \times 10^{-09}$) | $3.21 \times 10^{-06}$ ($1.90 \times 10^{-06}$ - $4.84 \times 10^{-06}$) |
| 2 | 1228 | $9.47 \times 10^{-10}$ ($3.70 \times 10^{-10}$ - $1.70 \times 10^{-09}$) | $1.16 \times 10^{-06}$ ($4.54 \times 10^{-07}$ - $2.09 \times 10^{-06}$) |
| 3 | 1267 | $1.18 \times 10^{-09}$ ($6.85 \times 10^{-10}$ - $1.77 \times 10^{-09}$) | $1.49 \times 10^{-06}$ ($8.68 \times 10^{-07}$ - $2.24 \times 10^{-06}$) |
| 4 | 2504 | $1.04 \times 10^{-09}$ ($6.85 \times 10^{-10}$ - $1.39 \times 10^{-09}$) | $2.61 \times 10^{-06}$ ($1.72 \times 10^{-06}$ - $3.47 \times 10^{-06}$) |
| 5 | 2903 | $1.11 \times 10^{-09}$ ($5.97 \times 10^{-10}$ - $1.77 \times 10^{-09}$) | $3.21 \times 10^{-06}$ ($1.73 \times 10^{-06}$ - $5.15 \times 10^{-06}$) |
| 7 | 2761 | $9.35 \times 10^{-10}$ ($5.97 \times 10^{-10}$ - $1.34 \times 10^{-09}$) | $2.58 \times 10^{-06}$ ($1.65 \times 10^{-06}$ - $3.69 \times 10^{-06}$) |
| 8 | 670 | $7.08 \times 10^{-10}$ ($1.22 \times 10^{-10}$ - $1.62 \times 10^{-09}$) | $4.74 \times 10^{-07}$ ($8.19 \times 10^{-08}$ - $1.08 \times 10^{-06}$) |
| 9 | 1789 | $5.59 \times 10^{-10}$ ($2.81 \times 10^{-10}$ - $8.45 \times 10^{-10}$) | $1.00 \times 10^{-06}$ ($5.03 \times 10^{-07}$ - $1.51 \times 10^{-06}$) |
| 10 | 2006 | $8.00 \times 10^{-10}$ ($4.42 \times 10^{-10}$ - $1.21 \times 10^{-09}$) | $1.60 \times 10^{-06}$ ($8.86 \times 10^{-07}$ - $2.42 \times 10^{-06}$) |
