# Supplementary material for: How strong was the bottleneck associated to the peopling of the Americas? New insights from multilocus sequence data
Source: Genet Mol Biol. 2018;41(1 Suppl 1):206–14. doi: 10.1590/1678-4685-GMB-2017-0087 (PMC5913727; doi:10.1590/1678-4685-GMB-2017-0087)
Supplement: Supplementary file 9 [file 1415-4757-GMB-41-01-2017-0087-s007.pdf]

Supplementary Material to "How strong was the bottleneck associated to the peopling of the Americas? New insights from multilocus sequence data"

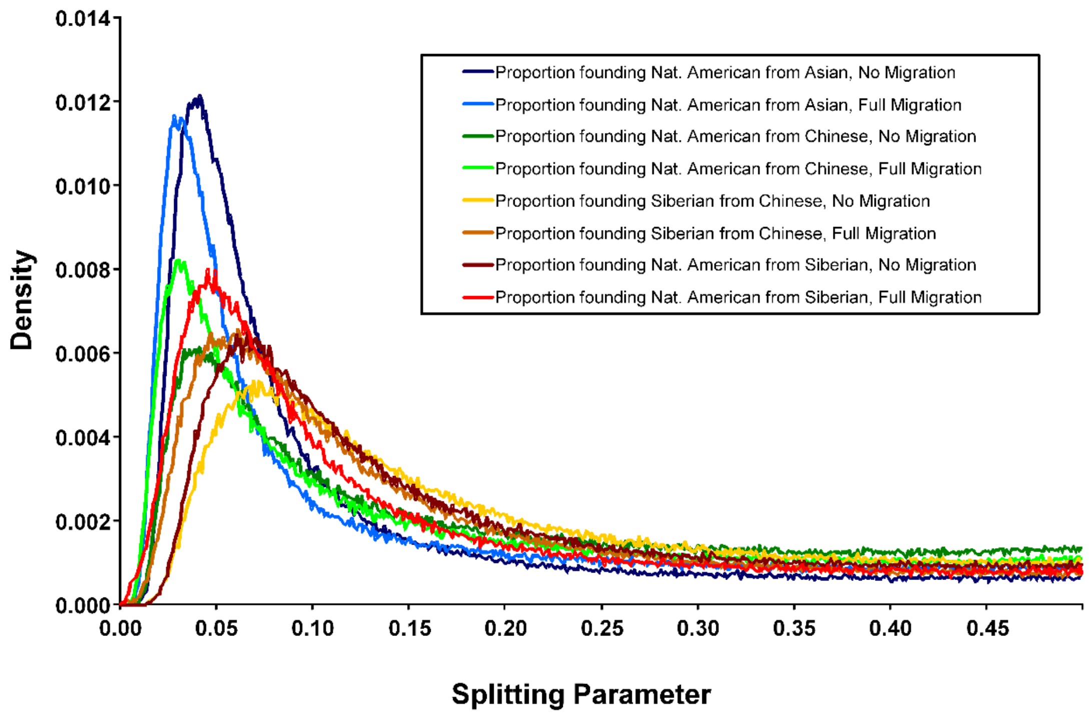

Figure S7 – Posterior density for the splitting parameter s for all scenarios.
